# Drug Repurposing for the Treatment of Acute Myeloid Leukemia

**DOI:** 10.3389/fmed.2017.00211

**Published:** 2017-11-29

**Authors:** Vibeke Andresen, Bjørn T. Gjertsen

**Affiliations:** ^1^Center for Cancer Biomarkers (CCBIO), Department of Clinical Science, Precision Oncology Research Group, University of Bergen, Bergen, Norway; ^2^Department of Internal Medicine, Hematology Section, Haukeland University Hospital, Bergen, Norway

**Keywords:** drug repurposing, drug repositioning, biomarkers, cancer, acute myeloid leukemia, therapy development

## Abstract

Acute myeloid leukemia (AML) is a heterogeneous disease characterized by the accumulation of immature myeloid progenitor cells in the bone marrow, compromising of normal blood cell production and ultimately resulting in bone marrow failure. With a 20% overall survival rate at 5 years and 50% in the 18- to 65-year-old age group, new medicines are needed. It is proposed that development of repurposed drugs may be a part of the new therapy needed. AML is subdivided into recurrent molecular entities based on molecular genetics increasingly accessible for precision medicine. Novel therapy developments form a basis for novel multimodality therapy and include liposomal daunorubicin/cytarabine, broad or FLT3-specific tyrosine kinase inhibitors, Bcl-2 family inhibitors, selective inhibitors of nuclear export, metabolic inhibitors, and demethylating agents. The use of non-transplant immunotherapy is in early development in AML with the exceptional re-approval of a toxin-conjugated anti-CD33. However, the full potential of small molecule inhibitors and modalities like immunological checkpoint inhibitors, immunostimulatory small molecules, and CAR-T cell therapy is unknown. Some novel therapeutics will certainly benefit AML patient subgroups; however, due to high cost, more affordable alternatives are needed globally. Also the heterogeneity of AML will likely demand a broader repertoire of therapeutic molecules. Drug repurposing or repositioning represent a source for potential therapeutics with well-known toxicity profiles and reasonable prices. This implies that biomarkers of response need to accompany the development of antileukemic therapies for sharply defined patient subgroups. We will illustrate repurposing in AML with selected examples and discuss some experimental and regulatory limitations that may obstruct this development.

## Introduction

**Figure 1:**
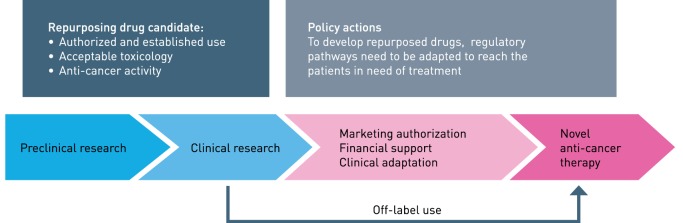
Repurposing drugs to anticancer therapy. The different phases indicated. The term “financial orphan” has been coined for repurposing registrated off-patent molecules for new diseases. Clinical trials represent a substantial cost, mainly because of regulatory precursions and careful validation of data. Figure modified from Ref. (12).

Repurposing is a recognized strategy in drug discovery and development where an already approved drug is used for diseases other than originally indicated (Figure 1) [for reviews, see Ref. (1–4)]. The development of a novel drug takes between 13 and 15 years from bench-to-bedside, where the estimated cost from research to a marketing approval is between 2 and 3 billion US dollars (5). In addition, drug development is associated with a high failure rate and most drugs never reach a final Food and Drug Administration (FDA) approval. This is highly illustrated by comparing the number of FDA approved drugs in 1976 and 2014, being 26 and 41, respectively. However, the cost of drug discovery has increased more than 15-fold since 1975 (5). From a global perspective, a successfully developed drug will be priced high and consequently becomes inaffordable for the majority of the patients in need. The timeframe of repurposing an already approved drug has been estimated to be approximately 6 years, because initial drug development have already been performed and have a cost of 300 million US dollars, in the end, resulting in much more affordable drugs for the patients (5). Repurposing is often associated with discovery of a new mechanism of action and elucidation of a novel molecular target (6–8). Screening techniques directed toward new diseases and mechanisms are central to a repurposing strategy together with systems biology approaches that have rejuvenated the field (9). Recent development in advanced disease models *ex vivo* and *in vivo* may be essential for successful development. Additionally, in cancer prevention, there are several examples of repurposing (10, 11). However, developments both in prevention therapy and direct therapy are moving slowly. Tumor heterogeneity and cancer subtypes are suggested as important reasons for limited overall benefit.

### Why Repurposing Drugs?

Repurposing is fruitful because we already know the spectrum of side effects, and how these drugs are administered and distributed in the organism (3, 4). We now understand that most small molecule substances may have more than one molecular target. Evaluation assays of new therapeutic targets are essential for success. The drug concentration required to achieve a desired pharmacological effect could vary greatly. Consequently, if a substantially higher drug concentration is required for a new pharmacological effect, a new preclinical evaluation regarding absorption, distribution, metabolism, and excretion will be necessary. Clinical evaluation of toxicity and side effects would also have to be re-evaluated.

### A Job to Be Done: The Need of New Medicines in Cancer Therapy

The major challenge of cancer therapy today is the therapeutic effect in metastatic cancer or surgically non-resectable tumors. Five years mortality above 90% in severe metastatic cancer and particular aggressive cancers like pancreatic cancer and cytogenetically defined high-risk acute myeloid leukemia (AML) underscores an urgent need for new drugs in cancer treatment. Likewise, refractory or relapsing disease is therapeutically challenging and demands new drugs. These new drugs should reflect the emerging knowledge of genetics, molecular characterization of tumors, and various tumor–host interactions (13). A striking trend in cancer diagnostics is the subdivision of particular diseases, from morphological and anatomical–pathological classification to disease subsets characterized by recurrent mutations (14). To some degree, mutations in similar genes in different cancers can be treated with the same therapy, for example, TKIs in BCR-ABL1-positive AML (15), acute lymphocytic leukemia (ALL) (16), and chronic myeloid leukemia (17, 18). A similar trend in *TP53* mutated cancers could be plausible through small molecules proposed to activate the mutated p53 protein (19). A deeper understanding of tumor cell clonality and clonal evolution has followed increasing understanding of disease heterogeneity (9). Clonal plasticity is frequently present both during both disease development and during cancer therapy. The clonal repertoire in one patient may, therefore, need a wider selection of molecules able to target various cellular mechanisms driving the tumor cells. Finally, the tumor–host interaction maybe essential to obtain disease control and to provide control of clonal evolution (20).

Surprisingly, among the approximately 1,600 FDA-approved drugs and drugs in late clinical development, the number of druggable molecules in a cell or organ is estimated to be 670 of the 21,000 genes and 1.5 million proteins and isoforms expressed (21). If these approved drugs could be exploited across diseases and particularly in cancer therapy, the therapeutic toolbox would be significantly expanded.

**Figure 2:**
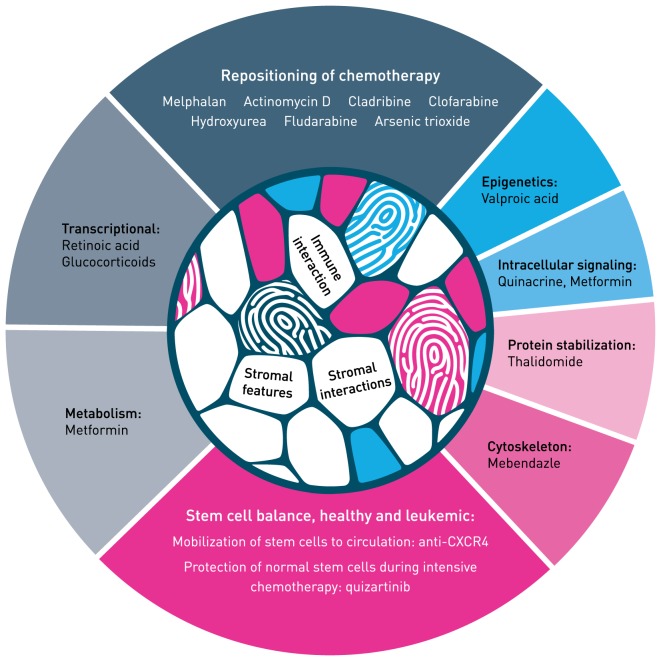
Hallmark of repurposing in acute myeloid leukemia. Main classes of mechanism are presented with selected examples. Both repositioning of chemotherapy (top of figure) and repurposing of non-cytotoxic medicines (sides and bottom) need identification of biomarkers that identify patients who respond to therapy. For more examples on repurposing in blood cancer, see Ref. (22). The mechanism of action of most therapeutic molecules is incompletely understood in the context of tumor–host interaction, both for stromal interaction and immune responses. The tumor–host interaction illustrated in the inner circle is modified from Centre of Cancer Biomarkers (www.ccbio.no). Figure modified from Ref. (4) and inspired by Hallmark of Cancer, by Hanahan and Weinberg (13).

We will present selected examples of repurposing in AML and aspects of the process that may be of importance for further development in this aggressive blood cancer (Figure 2).

Repurposing and repositioning are often used synonymous in drug development. We suggest using the term repositioning for approved and established chemotherapeutics further developed for use in AML, either added to intensive induction therapy or in a new therapy combination or sequence. Repurposing will be used to describe agents used in non-cancer diseases, like infectious, cardiovascular, metabolic, or convulsive diseases, and how these agents further could be developed into antileukemic medicines. We will present a selection of repurposing examples that completed the translation from bench to bedside and some examples that are in development. Possible hindrances and how these may be dealt with will also be discussed (Figure 1).

### AML—Treatment and Survival

Acute myeloid leukemia is an aggressive blood cancer derived from myeloid hematopoietic stem-like cells where recurrent mutations and cytogenetic features predict therapy response and risk for relapse (14). Cytogenetic aberrancies with multiple monosomies indicate less than 5% 5-year survival in AML patients between 18 and 65 years of age (23), while special chromosomal translocations such as the 15;17 in the acute promyelocytic leukemia (APL; AML M3), involving the retinoic acid receptor, suggest more than 90% 50 months survival and may be treated chemotherapy-free with all-trans retinoic acid and arsenic trioxide (24). The history of therapy development in APL is unique due to its close ties to Chinese traditional medicine, where both all-trans retinoic acid and arsenic trioxide have their origin (25). The recurrent mutations in receptor tyrosine kinase FLT3 and nucleophosmin 1 (NPM1) can be used together in patients without cytogenetic aberrations and thereby stratify 5 years survival between 9% (FLT3-ITD mutated, NPM1 wild type) and 61% (NPM1 mutation, FLT3 wild type) (14). This guides the choice of allogeneic stem cell transplantation as consolidation therapy. However, stem cell transplantation has up to 20% treatment-related mortality and about 20% of the patients may not be offered this therapy due to comorbidity (26). Paradoxically, the immediate choice of therapy at diagnosis is not translated into clinical practice per 2017 except for the approximately 10% of patients with translocation 15;17 treated with retinoic acid. The high risk nature for early death in APL (27) has led to international guidelines advising immediate medication with all-trans retinoic acid based on immediate suspicion by blood and bone marrow cytology. In the remaining AML subsets, new drugs are slowly emerging (14). New effective drugs targeting FLT3 signaling, metabolism (IDH1/2), and cell death (Bcl-2) are currently approved or will likely be approved based on recent phase III clinical trials addressing molecular stratification in therapy guidance (28).

## Repositioning of Chemotherapy for AML

Repositioning of chemotherapy to AML may have a significant potential if we precisely were capable of selecting responders and avoiding non-responders (Figure 2). The combination of cytarabine and anthracyclines has been the gold standard treatment of AML patients for decades (14, 28). There are partially historical reasons for certain chemotherapeutics being excluded from use in AML. Several agents have been introduced in extensive therapy development programs, however, unfortunately, because of lack of effect or only weak beneficial effect in subgroups of patients, their final position in AML is not determined. Many of the antimetabolites appear to have striking efficacies in up to 30% of the patients (29). If accurate biomarkers of response were available, our toolbox would immediately be filled with easy-to-use low-cost agents. We could speculate that repositioning in the near future will be catalyzed by next-generation AML disease profiling using novel immune mapping, ultra-deep sequencing, epigenetic characterization, or sensitive quantitative proteomics. Determining the complete responders upfront of low-dose chemotherapy may be invaluable for elderly unfit AML patients.

### Cladribine

Cladribine (2-chloro-2′-deoxyadenosine; Leustatin) is a synthetic purine nucleoside antimetabolite that is highly effective treatment for the B-cell disease hairy cell leukemia; curing more than half of patients treated. Cladribine is an analog of deoxyadenosine, resistant to deaminase, and first synthesized in the beginning of the 1980s for investigation of antileukemic and antilymphocytic activity (30, 31). This was based on the observations that patients with a genetic defect of adenosine deaminase essential for the conversion of lymphotoxic deoxyadenosine to non-toxic deoxyinosine showed lymphopenia and severe immunodeficiency (32). Recently, it has been tested in combination with anthracycline and cytarabine in fit patients with *de novo* AML (33). This triplet combination is one of the few enhancement of intensive AML therapy that improves overall survival. We speculate that cost is why cladribine is not used routinely in most of the Western world. Poland has reasonable priced production of cladribine, while most Western European countries have highly priced supplies. This exemplifies that off-patent drugs not necessarily are cheap but could experience significant cost increases (34), e.g., a price dictated by production limitations. Therefore, it is not likely that agents like cladribine will be included in international guidelines in combination with intensive chemotherapy. Approval of FLT3-targeting midostaurine and fast-track development programs of drugs targeting IDH1/IDH2 will most likely be added to the current intensive therapy, possible with enhanced effect and less adverse events.

### Clofarabine

Clofarabine (2-chloro-2′-fluoro-2′-deoxyarabinosyladenine) is a second-generation analog of 2′-deoxyadenosine, synthesized in 1992 and approved in 2004 for treatment of children with refractory or relapsed ALL (35). It was evident early on that clofarbine was effective against AML (36). Recently, the HOVON/SAKK Group reported that clofarabine did not provide a benefit in overall survival of newly diagnosed AML patients in the age group of 18–65 years (37). However, in the intermediary risk group, a clinical benefit was observed. This underscores the potential benefit in certain AML subsets and that the search for molecular markers of response should be intensified.

Cladribine and clofarabine belong to a large family of nucleoside and nucleobase antimetabolites with great importance in treatment of cancer and infections (38). Many of these molecules inhibit ribonucleotide reductase (39), likely an important component of the antileukemic effect. Fludarbine is per orally formulated and registered for combination use in AML (16, 40), but both cladribine and clofarabine may well have similar or even better therapeutic effect. Our understanding is that until feasible biomarkers are available, the justified position of cladribine and clofarabine will not be clarified in AML.

### Actinomycin D

Actinomycin D (dactinomycin, Cosmegen) was described in 1940 as the first antibiotic demonstrating anticancer effect (41). It interacts directly with DNA and blocks RNA synthesis by inhibiting RNA polymerase I, II, and III; specifically inhibiting RNA polymerase I activity only at low concentrations (42, 43). Actinomycin D has been approved for use in rare cancers such as Wilms tumor, rhabdomyosarcoma, Ewing’s sarcoma, trophoblastic neoplasm, testicular cancer, and certain types of ovarian cancer (44, 45). Recently, actinomycin D at low doses was reported effective when treating unfit AML with *NPM1* mutations (46). This is intriguing because of its limited toxicity and the fact that one-third of all AML patients have a NPM1 mutation (47). Mechanistically, actinomycin D has been shown to affect ribosomal biogenesis and thereby altering pre-mRNA splicing of tumor suppressor p53 (48). Mutations in the pre-mRNA splicing machinery is a feature associated with AML, and NPM1 itself play an important role in ribosomal biogenesis. Interestingly, AML with mutated NPM1 appear to have relative increase in the p53 spliced isoforms beta and gamma (49). Finally, the myeloid AML blasts have a high level synthesis of ribosomes and may, therefore, be particular susceptible to actinomycin D (50).

### Melphalan

Melphalan is an alkylating agent used in a wide dose span, from treatment of unfit multiple myeloma to bone marrow eradication in autologous stem cell transplantation. Low-dose melphalan has repeatedly been described to benefit selected patients with myelodysplasia and AML. An early report described that 2 mg daily orally administrated melphalan-induced remission in myelodysplasia refractory anemia with excess blasts and in transformation to AML (51). A single arm clinical study of 21 patients with AML indicated 30% complete responses and 10% partial responses when treated for 4–6 weeks (29). Responses lasted 12–55 weeks, and relapses responded to rechallenge with melphalan. Interestingly, patients with normal karyotype and low bone marrow cellularity were the most frequent responders. Two cases describing relapses on melphalan demonstrated clonal evolution with appearance of the high-risk loss of 17p including the *TP53* gene (52). Both of these patients were resistant to cytarabine, the standard drug used extensively in AML. Based on the knowledge that melphalan induces DNA damage, melphalan is not ideal for treatment of younger patients. Melphalan-induced genomic instability in particularly susceptible patients may be the reason for secondary hematological malignancies, found in a subset of melphalan-treated myeloma patients (53, 54). Understanding better, the genomic susceptibility to melphalan and other antimetabolites may facilitate safer usage of these agents.

### Hydroxyurea

Hydroxyurea is authorized as a drug to decrease the level of white blood cells in AML, but it has never been shown to improve survival compared to best supportive therapy (55). Hydroxyurea is regarded as a safe agent not inducing DNA damage through its extensive use in younger patients with hemoglobinopathies (56, 57). Interestingly, hydroxyurea provides antileukemic effects in novel combinations. We have preclinically tested hydroxyurea in combination with valproic acid and experienced effects on DNA repair unique for the combination (58). This combination is apparently well tolerated in unfit AML patients (59), but data from controlled trials are lacking.

### Arsenic Trioxide Repositioned for Non-APL

Arsenic trioxide combined with all-trans retinoic acid is close to a standard therapy for patients with t(15;17) M3 AML (60). However, for AML (non-APL), arsenic trioxide in combination with low-dose cytarabine or standard induction therapy have not been successful (61–64). These clinical observations are disappointing in light of a wide range of preclinical studies that indicate potential combinations with specific mechanisms of action.

## Repurposing Non-Chemotherapy Agents for AML

The delineation of chemotherapeutics and many non-chemotherapeutics could be artificial. If doses are increased, most therapeutic molecules will induce cytotoxicity. However, we will discuss agents that are regarded non-toxic when used at approved doses. Lessons learned from all-trans retinoic acid in APL (AML M3), as well as new molecules with strong pro-differentiating effects, indicate just how cells are brought out of their proliferative state matters for therapy response. Furthermore, several agents appear to show low toxicity by themselves, but alter the threshold for undergoing cell death when exposed to additional antileukemic therapy. A general concept of non-chemotherapy agents, including all-trans retinoic acid in APL, seems to be that monotherapy usage is not feasible.

### Glucocorticoids in AML

Glucocorticoids in AML, so far, play no therapeutic antileukemic role, and its use is actually discouraged due to an immunosuppressive effect. However, two reports suggest that glucucorticoids may be beneficial in AML subsets. Patients resistant to cytarabine and with wild-type FLT3 may respond to glucocorticoids (65). More specifically, an entity of AML patients with loss-of-function mutations in the *RUNX1* gene is proposed to have particular benefit of glucocorticoids apparently in a *RUNX1* dose-dependent way (66). A biomarker for glucocorticoid use may be implemented quickly in routine therapy of AML, particularly since glucocorticoids are already routinely used in the treatment of ALL.

### Per Oral Antidiabetics

Per oral antidiabetics are proposed to have a role in AML therapy, particularly based on awareness of metabolism as an important mechanism in AML cells. The oral hypoglycemic metformin activates the adenosine monophosphate-activated protein kinase pathway dependent on the tumor suppressor LKB1. The LKB1/AMPK pathway is negatively activated by ERK in AML cells (67), and ERK is activated in the 50% of AML cases with mutations in intracellular signal transduction pathways (68, 69). Particularly, FLT3-ITD mutated AML cases may be susceptible for metformin and maybe with enhanced efficiency when combined with the kinase inhibitor sorafenib or the experimental drug 6-benzoylthioinosine (70, 71). Experiments in animal model xenografts of colon cancer and prostate cancer have demonstrated tumor suppressive effects of metformin (72, 73). Further, registry studies of diabetic patients on metformin have indicated reduced risk of pancreatic cancer (74). Based on this and other experimental evidence, a clinical trial in relapsed/refractory AML has been proposed combining metformin and cytarabine (https://ClinicalTrials.gov Identifier: NCT01849276).

### Statins, HMG-CoA Reductase Inhibitors

Statins, HMG-CoA reductase inhibitors, have been explored as antileukemic agents in AML. Statins tested as monotherapy in clinical trials have not shown convincing results. Statins may exemplify the limited potential of monotherapy of a repurposed drug in an aggressive blood cancer like AML. Current understanding of the dosing of statins is not complete (75, 76) and likely needs careful modeling for optimal effects of statins in AML. Two reports suggest that pravastatin may be beneficial in combination with idarubicin and cytarabin in relapsed AML, while *de novo* AML did not benefit from this combination (75, 76). These clinical trials combining statins with other agents have demonstrated encouraging results. Interestingly, experimental evidence indicates immunomodulatory properties, including increased tumor infiltration of effector CD8 T-cells and M2-like tumor-associated macrophages (77). These and novel combinations need to be further developed.

### The Benzimidazole Family

The benzimidazole family of antihelmintic drugs have been evaluated for antileukemic effects. The benzimidazoles are suggested to inhibit amino peptidase activity and glutamate catabolism, reduce glucose uptake, increase intracellular calcium levels, and inhibit microtubule formation [for references, see Ref. (78)]. The antileukemic activity of mebendazole was discovered in an *in vitro* drug screen of AML cells with genetic alterations in Mixed Lineage Leukemia (79). Leukemia cell lines in the NCI-60 panel including HL-60, K562, and CEM were sensitive to mebendazol *in vitro* (80). More in-depth characterization of the antileukemic effect of flubendazole demonstrates altered microtubule function and mitotic catastrophe with a binding site on tubulin that differed from Vinca alkaloids tubulin inhibitors (78). The synergism of flubendazole with Vinca alkaloid vinblastine in the cell line OCI-AML2 and in a leukemia xenograft animal model may reflect the different tubulin-binding sites of flubendazole and vinblastine. To this date, no clinical trials in AML have been initiated using these antihelmintic agents.

### Thalidomide: Development of a New Class of Targeted Molecules against Blood Cancer

Thalidomide was developed as a sedative and antiemetic in the late 1950s, and its use by pregnant women resulted in a catastrophic occurrence of birth defects and stillborn. Thalidomide was withdrawn, but its efficiency in severe inflammatory skin disorders and in Hansen’s disease (erythema nodosum leprosum) justified a limited use. Promoted by patient advocacy groups; thalidomide was tested in the plasma cell malignancy multiple myeloma nearly 50 years after its withdrawal, identifying responders in up to 20% of the patients (81). In AML and myelodysplasia syndromes (MDS), thalidomide induced a clinical response of 25 and 56%, respectively (82, 83). Recently, the combination of thalidomide and azacitidine was demonstrated tolerable in a phase II clinical trial in patients with clinically advanced MDS, chronic myelomonocytic leukemia, and low-blast count AML (84). But it took more than 60 years before a more complete understanding of the molecular mechanism emerged (7). Explaining both the teratogenicity and its antitumor response, the inhibitory action on the E3 ubiquitin ligase cereblon was discovered. Several more efficient thalidomide analogs were already in development based on the clinical effect. One such analog, lenalidomide, has shown to be particular effective in myelodysplasia lacking one copy of the 5q chromosome. Among several genes affected, 5q deletion results in haploinsufficency of casein kinase 1, and lenalidomide targeting of cereblon results in devastating low level of casein kinase 1 and subsequently tumor cell death (85). Myelodysplasia with 5q minus and response to lenalidomide is an example of synthetic lethality that could be necessary in order to make repurposing feasible for patients. It is likely that development of more molecules targeting the E3 ubiquitin ligase cereblon and its related pathways may provide us with efficient therapeutic targets.

### Valproic Acid: From Anticonvulsant to Antileukemic

Valproic acid (n-dipropylacetic acid, 2-propylvaleric acid, or 2-propylpentanoic acid) derived from valeric acid naturally produced by the flowering plant valarian (*valeriana officinalis*) was first synthesized by Beverly S. Burton in 1882 (86). This branched short-chain fatty acid was used as a physiologically inert solvent for organic compounds for nearly a century before the 1963 discovery that valproic acid had an anticonvulsant activity on its own (87). Clinically, valproic acid (Orfiril, Deprakine, Depakote, Convulex, Epilim, Stavzor) is used today as an long-term treatment of anticonvulsant in epilepsy and mood stabilizing drug for bipolar disorder (88). Valproic acid is administered orally with available routine measurements of serum levels and has a low toxicity profile. In 2001, valproic acid was rediscovered for its anticancer activity as a histone deacetylase (HDAC) inhibitor and importantly also found to induce differentiation and/or apoptosis of transformed hematopoietic stem cells and AML cells from patients (89–91). DNA hypoacetylation of histone-associated proteins leads to tight chromatin packaging resulting in repression of genes involved in differentiation, proliferation, and apoptosis. HDACs are often overexpressed in cancer cells, also in AML cells (92). Preclinical studies showed enhanced differentiation and apoptosis in AML cells when combining valproic acid with other drugs (59, 93–95).

Valproic acid is currently involved in many different anticancer clinical trials (88). Furthermore, valproic acid often enhance or synergize with a numerous of drugs (96). Recently, valproic acid was combined with standard induction therapy in elderly AML patients, and although not resulting in an overall improved clinical outcome, the 5-year relapse-free survival was significantly increased for the patients additionally treated with valproic acid (97). Interestingly, the AML patients that particularly benefited from the additional valproic acid treatment were NPM1-mutated (97).

We examined this antileukemic effect of valproic acid in AML in combination with all-trans retinoic acid and theophylline, aiming for a combined epigenetic, transcriptional, and signaling transduction effect that resulted in increased differentiation and programmed cell death of tumor cells (98, 99). Our and others low-dose combination approach indicate 8–20% responders, with beneficial effect on low platelet levels. Several combinations of valproic acid with new and old drugs appear attractive, like the well-tested antimetabolite hydroxyurea and the new class MDM2 inhibitor nutlin-3 (58, 95). Novel low-toxic combinations with valproic acid are in development and may be feasible to test in patients. However, the ability to perform substantial clinical trials with valproic acid that secure indication in cancer is challenging.

### Quinacrine (Mepacrine, Atabrine)

Quinacrine (mepacrine, Atabrine) is an acridine dye developed in the US as an antimalarial agent at the beginning of the Second World War and used by millions of military personnel. This allowed for extensive registration of side effects and toxicity of three million soldiers taking quinacrine for up to four years. (100). The toxicology profile is well known, dominated by gastrointestinal symptoms, dizziness and nausea, exanthema, and at doses, above 500 mg daily reversible psychosis may occur. Quinacrine is currently used off-label for diseases such as therapy refractory giardiasis and as therapy enhancer in systemic lupus erythematosus (100). The mechanisms of action for quinacrine have been extensively explored together with other acridine molecules. The DNA intercalating property of quinacrine is not associated with DNA damage, in contrast to the anthracycline chemotherapeutics, but has been used to stain chromosomes for G-banding where placental transfer of leukemic cells from fetus to mother was demonstrated. Early works also indicated that quinacrine accumulates in tumor tissue in mouse and rat models, including leukemic spleen (100). Mechanisms of action include inhibition of the NF-κB signal transduction pathway and activation of tumor suppressor protein p53 (6, 101). Quinacrine was recently selected as the number one hit in a drug screen searching for compounds with high antileukemic activity and low toxicity toward healthy peripheral mononuclear cells where ribosome biogenesis was found to be the target of quinacrine (102). Thus, ribosome biogenesis inhibition will result in the release of ribosomal proteins binding to the E3 ubiquitin ligase MDM2, thereby blocking p53 ubiquitination. The result implies that quinacrine indirectly inhibit MDM2, thereby stabilizing p53 protein that induces p53-dependent gene expression (6). AML is a disease where 90% of the patients have non-mutated *TP53*, and the effect of quinacrine on MDM2 is strongest in these patients. Increased activation of the NF-κB pathway is known in AML (103, 104) and is suggested to induce antiapoptotic Bcl-2 in the leukemic cells, causing a higher threshold for undergoing chemotherapy induced cell death.

### Novel Mechanisms with Therapeutic Implications

Quizartinib is a promising FLT3-targeted kinase inhibitor in late development for AML (105). The role of FLT3 and the FLT3 ligand is known in some detail, e.g., in aplasia, the FLT3 ligand is highly increased in blood and bone marrow. A novel use of quizartinib is suggested through the use for preventing chemotherapeutic myelosuppression (106). Myelosuppression is regarded as a natural and unsuspendable process in intensive AML therapy. An interesting feature about mutated FLT3-ITD signaling is based on the subcellular localization of the mutated FLT3 to the endoplasmic reticulum, not typical for wild-type FLT3 signaling (107–109). Based on this difference in signaling between wild-type and FLT3-ITD, normal and leukemic stem cells may respond differently to FLT3 inhibitor treatment. By protecting normal hematopoietic stem cells from FLT3 ligand signaling, the myeloablasive phase could be significantly shortened.

Bromocriptine is a dopamine receptor agonist/antagonist used to treat Parkinson’s disease, acromegaly, galactorrhea, hyperprolactinemia, and lately repositioned for diabetes mellitus. Bromocriptine was recently identified in a screen of FDA-approved drugs searching for drugs with potential of differentiation of AML cells (110). Serotonin receptor agonists are frequently used in the treatment of depression and migraines. Interestingly, the serotonin receptor type 1 (HTR1) was demonstrated expressed on AML cells and addition of HTR1 antagonist induced differentiation, increased cell death, and impaired clonogenic capacity (111).

Leukemic stem cells play a central role in relapsed and refractory AML and, therefore, developments of low-toxicity compounds targeting these cells are needed. Ongoing research is studying several compounds derived from natural sources such as plant extract from parthenolides with efficacy toward AML stem cells (112). We have recently reported antileukemic effects of the natural product avrainvillamide in AML with a strong antiproliferative activity and enhanced potency toward AML cells with NPM1 and FLT3 mutations (113, 114).

Leukemic progenitor cells are known to hide in stem cell niches of the bone marrow. In this situation, the leukemic cells are protected by stromal cells that directly or indirectly provide survival and protective factors that blunt the effect of chemotherapy (115). Inspired by the observation that antibody blockage of certain homing factors to the bone marrow niche, like CXCR4, may provide mobilization of hematopoietic stem cells, these blockers of niche homing have been proposed to mobilize leukemic cells before chemotherapeutic therapy (116). Several clinical trials currently address this concept (https://ClinicalTrials.gov IdentifierNCT01236144), and we are looking forward to data emerging from these trials on the effect on minimal residual disease and finally overall survival in AML.

## Challenges for Repurposing

Obviously, the experimental backbone of repurposing drugs in cancer therapy is challenging, followed by demanding clinical trials that need a sophisticated biomarker program for identifying therapy responders (Figure 1). If successful, the data obtained should solve the obstacle related to the approval mechanisms and regulations by medicines agencies. These hurdles could be solved if the development program has financial muscles, which is not very likely in projects emerging from smaller laboratories and academic institutions. The approval allows marketing at a specific indication listed on the drug’s label, and documentation including clinical trials that should lead to such approval is economically challenging. Furthermore, if the sale on a particular label or indication is limited, the producer may withdraw the marketing approval. In extreme examples, a drug (alemtuzumab, anti-CD52 against relapsed B-cell chronic lymphatic leukemia; CLL) was withdrawn from the initial cancer indication and reapproved for a larger market in multiple sclerosis (117, 118). At the same time, the company introduced the Campath Distribution Program (www.campathproviderportal.com) in the US to secure that eligible CLL patients got access to the drug free of charge. This exemption of anti-CD52 therapy illustrates that reimbursement of the drug by the patient’s governmental or private insurance agency will be based on the approval, and use of off-label therapy will be economically challenging.

## Emerging Technologies That Support Repurposing

System biology approaches has recently been used in a drug sensitivity test of AML cells from patients (9). Initiatives for advanced collections coupled with drug response mapping, e.g., gene expression and proteomics, will make development more rational and predictable (119). We have seen these developments be rewarding in orthotopic small animal models of AML, where collections of libraries of molecules coupled with advanced bioinformatics tools may improve the probability of discovering novel combinations of therapeutics authorized for other diseases than AML (120). More advanced *in silico* tools are on the brink to facilitate this development (121). Combination of miniaturization, microfluidics, and bioinformatics with artificial intelligence may represent the novel tools needed to answer the overdue question in chemotherapy repositioning: which patients will be optimal responders.

## Final Conclusion

At one end of the drug development spectrum, repurposing has the potential to bring old drugs rapidly into use for new diseases. At the other end, repurposing could serve to identify lead compounds being chemically modified in order to increase target affinity and reduce necessary dosing and toxicity. Novel formulations may add more dimensions to drug delivery and tissue targeting that make repurposing of medicines highly attractive. Widespread use of repurposing in therapy development may need regulatory steps to move forward, securing approved indications, safety data, and allowing insurance reimbursement. This needs international regulations that take into consideration the global need for cancer therapy. Cancer therapy needs a larger therapy toolbox and effective repurposing may be one important tool that increases the number of therapy responders in cancer.

## Author Contributions

Both authors contributed in writing and editing the manuscript.

## Conflict of Interest Statement

The authors declare that the research was conducted in the absence of any commercial or financial relationships that could be construed as a potential conflict of interest.

## References

[1] Sukhai MA Spagnuolo PA Weir S Kasper J Patton L Schimmer AD. New sources of drugs for hematologic malignancies. Blood (2011) 117:6747–55. 10.1182/blood-2011-02-315283 21511957 PMC3357971

[2] Gupta SC Sung B Prasad S Webb LJ Aggarwal BB. Cancer drug discovery by repurposing: teaching new tricks to old dogs. Trends Pharmacol Sci (2013) 34:508–17. 10.1016/j.tips.2013.06.005 23928289

[3] Pantziarka P Bouche G Meheus L Sukhatme V Sukhatme VP Repurposing drugs in your medicine cabinet: untapped opportunities for cancer therapy? Future Oncol (2015) 11:181–4. 10.2217/fon.14.244 25591833

[4] Sleire L Førde-Tislevoll HE Netland IA Leiss L Skeie BS Enger PØ Drug repurposing in cancer. Pharmacol Res (2017) 124:74–91. 10.1016/j.phrs.2017.07.013 28712971

[5] Nosenga N Can you teach old drugs new tricks? Nature (2016) 534:314–6. 10.108/534314a 27306171

[6] Gurova K. New hopes from old drugs: revisiting DNA-binding small molecules as anticancer agents. Future Oncol (2009) 5(10):1685–704. 10.2217/fon.09.127 20001804 PMC2821823

[7] Ito T Ando H Suzuki T Ogura T Hotta K Imamura Y Identification of a primary target of thalidomide teratogenicity. Science (2010) 327:1345–50. 10.1126/science.1177319 20223979

[8] de The H Le Bras M Lallemand-Breitenbach V. The cell biology of disease: acute promyelocytic leukemia, arsenic, and PML bodies. J Cell Biol (2012) 198:11–21. 10.1083/jcb.201112044 22778276 PMC3392943

[9] Pemovska T Kontro M Yadav B Edgren H Eldfors S Szwajda A Individualized systems medicine strategy to tailor treatments for patients with chemorefractory acute myeloid leukemia. Cancer Discov (2013) 3:1416–29. 10.1158/2159-8290.CD-13-0350 24056683

[10] Bains SJ Mahic M Myklebust TÅ Småstuen MC Yaqub S Dørum LM Aspirin as secondary prevention in patients with colorectal cancer: an unselected population-based study. J Clin Oncol (2016) 34:2501–8. 10.1200/JCO.2015.65.3519 27247217

[11] Heckman-Stoddard BM DeCensi A Sahasrabuddhe VV Ford LG. Repurposing metformin for the prevention of cancer and cancer recurrence. Diabetologia (2017) 60:1639–47. 10.1007/s00125-017-4372-6 28776080 PMC5709147

[12] Verbaanderd C Meheus L Huys I Pantziarka P. Repurposing drugs in oncology: next steps. Trends Cancer (2017) 3:543–6. 10.1016/j.trecan.2017.06.007 28780930

[13] Hanahan D Weinberg RA Hallmarks of cancer: the next generation. Cell (2011) 144:646–74. 10.1016/j.cell.2011.02.013 21376230

[14] Döhner H Estey E Grimwade D Amadori S Appelbaum FR Büchner T Diagnosis and management of AML in adults: 2017 ELN recommendations from an international expert panel. Blood (2017) 129:424–47. 10.1182/blood-2016-08-733196 27895058 PMC5291965

[15] Reboursiere E Chantepie S Gac AC Reman O. Rare but authentic Philadelphia-positive acute myeloblastic leukemia: two case reports and a literature review of characteristics, treatment and outcome. Hematol Oncol Stem Cell Ther (2015) 8:28–33. 10.1016/j.hemonc.2014.09.002 25300567

[16] Short NJ Kantarjian H Ravandi F Huang X Xiao L Garcia-Manero G A phase I/II randomized trial of clofarabine or fludarabine added to idarubicin and cytarabine for adults with relapsed or refractory acute myeloid leukemia. Leuk Lymphoma (2017) 18:1–8. 10.1080/10428194.2017.1349907 PMC5773400 28718728

[17] Steegmann JL Baccarani M Breccia M Casado LF Garcia-Gutierrez V Hochhaus A. European LeukemiaNet recommendations for the management and avoidance of adverse events of treatment in chronic myeloid leukaemia. Leukemia (2016) 30:1648–71. 10.1038/leu.2016.104 27121688 PMC4991363

[18] Radich JP Mauro MJ. Tyrosine kinase inhibitor treatment for newly diagnosed chronic myeloid leukemia. Hematol Oncol Clin North Am (2017) 31:577–87. 10.1016/j.hoc.2017.04.006 28673389

[19] Lehmann S Bykov VJ Ali D Andrén O Cherif H Tidefelt U Targeting p53 in vivo: a first-in-human study with p53-targeting compound APR-246 in refractory hematologic malignancies and prostate cancer. J Clin Oncol (2012) 30:3633–9. 10.1200/JCO.2011.40.7783 22965953

[20] McGranahan N Swanton C Clonal heterogeneity and tumor evolution: past, present, and the future. Cell (2017) 168:613–28. 10.1016/j.cell.2017.01.018 28187284

[21] Santos R Ursu O Gaulton A Bento AP Donadi RS Bologa CG A comprehensive map of molecular drug targets. Nat Rev Drug Discov (2017) 16:19–34. 10.1038/nrd.2016.230 27910877 PMC6314433

[22] McCabe B Liberante F Mills KI. Repurposing medicinal compounds for blood cancer treatment. Ann Hematol (2015) 94:1267–76. 10.1007/s00277-015-2412-1 26048243 PMC4488459

[23] Breems DA Van Putten WL De Greef GE Van Zelderen-Bhola SL Gerssen-Schoorl KB Mellink CH Monosomal karyotype in acute myeloid leukemia: a better indicator of poor prognosis than a complex karyotype. J Clin Oncol (2008) 26:4791–7. 10.1200/JCO.2008.16.0259 18695255

[24] Platzbecker U Avvisati G Cicconi L Thiede C Paoloni F Vignetti M. Improved outcomes with retinoic acid and arsenic trioxide compared with retinoic acid and chemotherapy in non-high-risk acute promyelocytic leukemia: final results of the randomized Italian-German APL0406 trial. J Clin Oncol (2017) 35:605–12. 10.1200/JCO.2016.67.1982 27400939

[25] Wang ZY Chen Z. Acute promyelocytic leukemia: from highly fatal to highly curable. Blood (2008) 111:2505–15. 10.1182/blood-2007-07-102798 18299451

[26] Kassim AA Savani BN Hematopoietic stem cell transplantation for acute myeloid leukemia: a review. Hematol Oncol Stem Cell Ther (2017). 10.1016/j.hemonc.2017.05.021 28666104

[27] Lehmann S Deneberg S Antunovic P Rangert-Derolf Å Garelius H Lazarevic V Early death rates remain high in high-risk APL: update from the Swedish Acute Leukemia Registry 1997-2013. Leukemia (2017) 31:1457–9. 10.1038/leu.2017.71 28232742

[28] Tamamyan G Kadia T Ravandi F Borthakur G Cortes J Jabbour E Frontline treatment of acute myeloid leukemia in adults. Crit Rev Oncol Hematol (2017) 110:20–34. 10.1016/j.critrevonc.2016.12.004 28109402 PMC5410376

[29] Denzlinger C Bowen D Benz D Gelly K Brugger W Kanz L. Low-dose melphalan induces favourable responses in elderly patients with high-risk myelodysplastic syndromes or secondary acute myeloid leukaemia. Br J Haematol (2000) 108:93–5. 10.1046/j.1365-2141.2000.01825.x 10651730

[30] Carson DA Wasson DB Beutler E. Antileukemic and immunosuppressive activity of 2-chloro-2’-deoxyadenosine. Proc Natl Acad Sci U S A (1984) 81:2232–6. 10.1073/pnas.81.7.2232 6585795 PMC345472

[31] Sigal DS Miller HJ Schram ED Saven A. Beyond hairy cell: the activity of cladribine in other hematologic malignancies. Blood (2010) 116:2884–96. 10.1182/blood-2010-02-246140 20634380

[32] Giblett ER Anderson JE Cohen F Pollara B Meuwissen HJ Adenosine-deaminase deficiency in two patients with severely impaired cellular immunity. Lancet (1972) 2:1067–9. 10.1016/S0140-6736(72)92345-8 4117384

[33] Pluta A Robak T Wrzesien-Kus A Katarzyna Budziszewska B Sulek K Wawrzyniak E Addition of cladribine to the standard induction treatment improves outcomes in a subset of elderly acute myeloid leukemia patients. Results of a randomized Polish Adult Leukemia Group (PALG) phase II trial. Am J Hematol (2017) 92:359–66. 10.1002/ajh.24654 28103640

[34] Khot UN Vogan ED Militello MA Nitroprusside and isoproterenol use after major price increases. N Engl J Med (2017) 377:594–5. 10.1056/NEJMc1700244 28792879

[35] Ghanem H Jabbour E Faderl S Ghandhi V Plunkett W Kantarjian H. Clofarabine in leukemia. Expert Rev Hematol (2010) 3:15–22. 10.1586/ehm.09.70 21082931

[36] Ghanem H Kantarjian H Ohanian M Jabbour E. The role of clofarabine in acute myeloid leukemia. Leuk Lymphoma (2013) 54:688–98. 10.3109/10428194.2012.726722 22957815 PMC5681218

[37] Löwenberg B Pabst T Maertens J van Norden Y Biemond BJ Schouten HC Therapeutic value of clofarabine in younger and middle-aged (18-65 years) adults with newly diagnosed AML. Blood (2017) 129:1636–45. 10.1182/blood-2016-10-740613 28049642

[38] Burke MP Borland KM Litosh VA. Base-modified nucleosides as chemotherapeutic agents: past and future. Curr Top Med Chem (2016) 16:1231–41. 10.2174/1568026615666150915111933 26369814

[39] Mannargudi MB Deb S. Clinical pharmacology and clinical trials of ribonucleotide reductase inhibitors: is it a viable cancer therapy? J Cancer Res Clin Oncol (2017) 143:1499–529. 10.1007/s00432-017-2457-8 28624910 PMC11819458

[40] Jabbour E Short NJ Ravandi F Huang X Xiao L Garcia-Manero G A randomized phase 2 study of idarubicin and cytarabine with clofarabine or fludarabine in patients with newly diagnosed acute myeloid leukemia. Cancer (2017) 123:4430–9. 10.1002/cncr.30883 28708931 PMC5739034

[41] Waksman SA Woodruff HB Bacteriostatic and bacteriocidal substances produced by soil actinomycetes. Proc Soc Exper Biol (1940) 45:609–14. 10.3181/00379727-45-11768

[42] Perry RP Kelley DE Inhibition of RNA synthesis by actinomycin D: characteristic dose-response of different RNA species. J Cell Physiol (1970) 76:127–39. 10.1002/jcp.1040760202 5500970

[43] Sobell MH. Actinomycin and DNA transcription. Proc Nat Acad Sci U S A (1985) 82:5328–31. 10.1073/pnas.82.16.5328 2410919 PMC390561

[44] Gaspar N Hawkins DS Dirksen U Lewis IJ Ferrari S Le Deley MC Ewing sarcoma: current management and future approaches through collaboration. J Clin Oncol (2015) 33:3036–46. 10.1200/JCO.2014.59.5256 26304893

[45] Lawrie TA Alazzam M Tidy J Hancock BW Osborne R First-line chemotherapy in low-risk gestational trophoblastic neoplasia. Cochrane Database Syst Rev (2016) 6:CD007102 10.1002/14651858.CD007102.pub4 PMC6768658 27281496

[46] Falini B Brunetti L Martelli MP Dactinomycin in NPM1-mutated acute myeloid leukemia. N Engl J Med (2015) 373:1180–2. 10.1056/NEJMc1509584 26376154

[47] Falini B Mecucci C Tiacci E Alcalay M Rosati R Pasqualucci L Cytoplasmic nucleophosmin in acute myelogenous leukemia with a normal karyotype. N Engl J Med (2005) 352:254–66. 10.1056/NEJMoa041974 15659725

[48] Choong ML Yang H Lee MA Lane DP Specific activation of the p53 pathway by low dose actinomycin D – a new route to p53 based cyclotherapy. Cell Cycle (2009) 8:2810–8. 10.4161/cc.8.17.9503 19657224

[49] Anensen N Hjelle SM Van Belle W Haaland I Silden E Bourdon JC Correlation analysis of p53 protein isoforms with NPM1/FLT3 mutations and therapy response in acute myeloid leukemia. Oncogene (2012) 31:1533–45. 10.1038/onc.2011.348 21860418

[50] Scala F Brighenti E Govoni M Imbrogno E Fornari F Treré D Direct relationship between the level of p53 stabilization induced by rRNA synthesis-inhibiting drugs and the cell ribosome biogenesis rate. Oncogene (2016) 35:977–89. 10.1038/onc.2015.147 25961931

[51] Omoto E Deguchi S Takaba S Kojima K Yano T Katayama Y Low-dose melphalan for treatment of high-risk myelodysplastic syndromes. Leukemia (1996) 10:609–14. 8618435

[52] Kerr R Cunningham J Bowen DT Low-dose melphalan in elderly acute myeloid leukaemia: complete remissions but resistant relapse with therapy-related karyotypes. Leukemia (2000) 14:953 10.1038/sj.leu.2401762 10803536

[53] Cuzick J Erskine S Edelman D Galton DAG A comparison of the incidence of the myelodysplastic syndrome and acute myeloid leukaemia following melphalan and cyclophosphamide treatment for myelomatosis. Br J Cancer (1987) 55:523–9. 10.1038/bjc.1987.107 3300761 PMC2001731

[54] Dispenzieri A Lacy MQ Greipp PR Multiple myeloma. In: Greer JP Foerster J Rodgers GM, editors. Wintrobe’s Clinical Hematology. Philadelphia, PA: Wolters Kluwer Health/Lippincott Williams & Wilkins (2009). p. 2417–8.

[55] Burnett AK Milligan D Prentice AG Goldstone AH McMullin MF Hills RK A comparison of low-dose cytarabine and hydroxyurea with or without all-trans retinoic acid for acute myeloid leukemia and high-risk myelodysplastic syndrome in patients not considered fit for intensive treatment. Cancer (2007) 109:1114–24. 10.1002/cncr.22496 17315155

[56] Steinberg MH McCarthy WF Castro O Ballas SK Armstrong FD Smith W The risks and benefits of long-term use of hydroxyurea in sickle cell anemia: a 17.5 year follow-up. Am J Hematol (2010) 85:403–8. 10.1002/ajh.21699 20513116 PMC2879711

[57] Hankins JS Aygun B Nottage K Thornburg C Smeltzer MP Ware RE From infancy to adolescence: fifteen years of continuous treatment with hydroxyurea in sickle cell anemia. Medicine (Baltimore) (2014) 93:e215. 10.1097/MD.0000000000000215 25526439 PMC4603125

[58] Leitch C Osdal T Andresen V Molland M Kristiansen S Nguyen XN Hydroxyurea synergizes with valproic acid in wild-type p53 acute myeloid leukaemia. Oncotarget (2016) 7:8105–18. 10.18632/oncotarget.6991 26812881 PMC4884979

[59] Fredly H Gjertsen BT Bruserud O. Histone deacetylase inhibition in the treatment of acute myeloid leukemia: the effects of valproic acid on leukemic cells, and the clinical and experimental evidence for combining valproic acid with other antileukemic agents. Clin Epigenetics (2013) 5:12. 10.1186/1868-7083-5-12 23898968 PMC3733883

[60] McCulloch D Brown C Iland H. Retinoic acid and arsenic trioxide in the treatment of acute promyelocytic leukemia: current perspectives. Onco Targets Ther (2017) 10:1585–601. 10.2147/OTT.S100513 28352191 PMC5359123

[61] Burnett AK Hills RK Hunter A Milligan D Kell J Wheatley K The addition of arsenic trioxide to low-dose Ara-C in older patients with AML does not improve outcome. Leukemia (2011) 25:1122–7. 10.1038/leu.2011.59 21475252 PMC6485444

[62] Wetzler M Andrews C Ford LA Tighe S Barcos M Sait SN Phase 1 study of arsenic trioxide, high-dose cytarabine, and idarubicin to down-regulate constitutive signal transducer and activator of transcription 3 activity in patients aged <60 years with acute myeloid leukemia. Cancer (2011) 117:4861–8. 10.1002/cncr.26097 21456022 PMC3129468

[63] Sekeres MA Maciejewski JP Erba HP Afable M Englehaupt R Sobecks R A Phase 2 study of combination therapy with arsenic trioxide and gemtuzumab ozogamicin in patients with myelodysplastic syndromes or secondary acute myeloid leukemia. Cancer (2011) 117:1253–61. 10.1002/cncr.25686 20960521

[64] Roboz GJ Ritchie EK Curcio T Provenzano J Carlin R Samuel M Arsenic trioxide and low-dose cytarabine in older patients with untreated acute myeloid leukemia, excluding acute promyelocytic leukemia. Cancer (2008) 113:2504–11. 10.1002/cncr.23855 18825661

[65] Malani D Murumägi A Yadav B Kontro M Eldfors S Kumar A Enhanced sensitivity to glucocorticoids in cytarabine-resistant AML. Leukemia (2017) 31:1187–95. 10.1038/leu.2016.314 27833094 PMC5420795

[66] Simon L Lavallee VP Bordeleau ME Krosl J Baccelli I Boucher G Chemogenomic landscape of RUNX1-mutated AML reveals importance of RUNX1 allele dosage in genetics and glucocorticoid sensitivity. Clin Cancer Res (2017). 10.1158/1078-0432.CCR-17-1259 28855357

[67] Kawashima I Mitsumori T Nozaki Y Yamamoto T Shobu-Sueki Y Nakajima K Negative regulation of the LKB1/AMPK pathway by ERK in human acute myeloid leukemia cells. Exp Hematol (2015) 43:524–33. 10.1016/j.exphem.2015.03.005 25846811

[68] Minami Y Yamamoto K Kiyoi H Ueda R Saito H Naoe T. Different antiapoptotic pathways between wild-type and mutated FLT3: insights into therapeutic targets in leukemia. Blood (2003) 102:2969–75. 10.1182/blood-2002-12-3813 12842996

[69] Chang F Steelman LS Lee JT Shelton JG Navolanic PM Blalock WL Signal transduction mediated by the Ras/Raf/MEK/ERK pathway from cytokine receptors to transcription factors: potential targeting for therapeutic intervention. Leukemia (2003) 17:1263–93. 10.1038/sj.leu.2402945 12835716

[70] Sabnis HS Bradley HL Tripathi S Yu WM Tse W Qu CK Synergistic cell death in FLT3-ITD positive acute myeloid leukemia by combined treatment with metformin and 6-benzylthioinosine. Leuk Res (2016) 50:132–40. 10.1016/j.leukres.2016.10.004 27760406 PMC5083204

[71] Wang F Liu Z Zeng J Zhu H Li J Cheng X Metformin synergistically sensitizes FLT3-ITD-positive acute myeloid leukemia to sorafenib by promoting mTOR-mediated apoptosis and autophagy. Leuk Res (2015) 39:1421–7. 10.1016/j.leukres.2015.09.016 26505133

[72] Nangia-Makker P Yu Y Vasudevan A Farhana L Rajendra SG Levi E Metformin: a potential therapeutic agent for recurrent colon cancer. PLoS One (2014) 9:e84369. 10.1371/journal.pone.0084369 24465408 PMC3896365

[73] Whitburn J Edwards CM Sooriakumaran P. Metformin and prostate cancer: a new role for an old drug. Curr Urol Rep (2017) 18:46. 10.1007/s11934-017-0693-8 28444639 PMC5405102

[74] Noto H Goto A Tsujimoto T Noda M. Cancer risk in diabetic patients treated with metformin: a systematic review and meta-analysis. PLoS One (2012) 7:e33411. 10.1371/journal.pone.0033411 22448244 PMC3308971

[75] Advani AS McDonough S Copelan E Willman C Mulford DA List AF SWOG0919: a Phase 2 study of idarubicin and cytarabine in combination with pravastatin for relapsed acute myeloid leukaemia. Br J Haematol (2014) 167:233–7. 10.1111/bjh.13035 25039477 PMC4188732

[76] Shadman M Mawad R Dean C Chen TL Shannon-Dorcy K Sandhu V Idarubicin, cytarabine, and pravastatin as induction therapy for untreated acute myeloid leukemia and high-risk myelodysplastic syndrome. Am J Hematol (2015) 90:483–6. 10.1002/ajh.23981 25689471 PMC4803479

[77] Mira E Carmona-Rodríguez L Tardáguila M Azcoitia I González-Martín A Almonacid L A lovastatin-elicited genetic program inhibits M2 macrophage polarization and enhances T cell infiltration into spontaneous mouse mammary tumors. Oncotarget (2013) 4:2288–301. 10.18632/oncotarget.1376 24317954 PMC3926827

[78] Spagnuolo PA Hu J Hurren R Wang X Gronda M Sukhai MA The antihelmintic flubendazole inhibits microtubule function through a mechanism distinct from Vinca alkaloids and displays preclinical activity in leukemia and myeloma. Blood (2010) 115:4824–33. 10.1182/blood-2009-09-243055 20348394

[79] Matchett KB Grishagin IV Kettyle LMG Gavory G Harrison T Mills KI Mebendazole: a candidate FDA approved drug for repurposing in leukaemia. Br J Haematol (2016) 173:5–178. 10.1111/bjh.14019 27073127

[80] Nygren P Fryknäs M Ågerup B Larsson R. Repositioning of the anthelmintic drug mebendazole for the treatment for colon cancer. J Cancer Res Clin Oncol (2013) 139:2133–40. 10.1007/s00432-013-1539-5 24135855 PMC3825534

[81] Landgren O Iskander K. Modern multiple myeloma therapy: deep, sustained treatment response and good clinical outcomes. J Intern Med (2017) 281:365–82. 10.1111/joim.12590 28205262

[82] Steins MB Padro T Bieker R Ruiz S Kropff M Kienast J. Efficacy and safety of thalidomide in patients with acute myeloid leukemia. Blood (2002) 99:834–9. 10.1182/blood.V99.3.834 11806984

[83] Steins MB Bieker R Padró T Kessler T Kienast J Berdel WE. Thalidomide for the treatment of acute myeloid leukemia. Leuk Lymphoma (2003) 44:1489–93. 10.3109/10428190309178769 14565649

[84] Kenealy M Patton N Filshie R Nicol A Ho SJ Hertzberg M. Results of a phase II study of thalidomide and azacitidine in patients with clinically advanced myelodysplastic syndromes (MDS), chronic myelomonocytic leukemia (CMML) and low blast count acute myeloid leukemia (AML). Leuk Lymphoma (2017) 58:298–307. 10.1080/10428194.2016.1190971 27268068

[85] Sperling AS Gibson CJ Ebert BL. The genetics of myelodysplastic syndrome: from clonal haematopoiesis to secondary leukaemia. Nat Rev Cancer (2017) 17:5–19. 10.1038/nrc.2016.112 27834397 PMC5470392

[86] Burton B On the propyl derivatives and decomposition products of ethylacetoacetate. Am Chem J (1882) 1882(3):385–95.

[87] Loscher W. Valproate: a reappraisal of its pharmacodynamic properties and mechanisms of action. Prog Neurobiol (1999) 58:31–59. 10.1016/S0301-0082(98)00075-6 10321796

[88] Chateauvieux S Morceau F Dicato M Diederich M. Molecular and therapeutic potential and toxicity of valproic acid. J Biomed Biotechnol (2010) 2010:479364. 10.1155/2010/479364 20798865 PMC2926634

[89] Göttlicher M Minucci S Zhu P Krämer OH Schimpf A Giavara S Valproic acid defines a novel class of HDAC inhibitors inducing differentiation of transformed cells. EMBO J (2001) 20:6969–78. 10.1093/emboj/20.24.6969 11742974 PMC125788

[90] Phiel CJ Zhang F Huang EY Guenther MG Lazar MA Klein PS. Histone deacetylase is a direct target of valproic acid, a potent anticonvulsant, mood stabilizer, and teratogen. J Biol Chem (2001) 276:36734–41. 10.1074/jbc.M101287200 11473107

[91] Blaheta RA Nau H Michaelis M Cinatl J Jr. Valproate and valproate-analogues: potent tools to fight against cancer. Curr Med Chem (2002) 9:1417–33. 10.2174/0929867023369763 12173980

[92] Tickenbrock L Klein HU Trento C Hascher A Gollner S Baumer N Increased HDAC1 deposition at hematopoietic promoters in AML and its association with patient survival. Leuk Res (2011) 35:620–5. 10.1016/j.leukres.2010.11.006 21176959

[93] Ferrara FF Fazi F Bianchini A Padula F Gelmetti V Minucci S Histone deacetylase-targeted treatment restores retinoic acid signaling and differentiation in acute myeloid leukemia. Cancer Res (2001) 61:2–7. 11196162

[94] Blaheta RA Michaelis M Driever PH Cinatl J Jr. Evolving anticancer drug valproic acid: insights into the mechanism and clinical studies. Med Res Rev (2005) 25:383–97. 10.1002/med.20027 15637697

[95] McCormack E Haaland I Venas G Forthun RB Huseby S Gausdal G Synergistic induction of p53 mediated apoptosis by valproic acid and nutlin-3 in acute myeloid leukemia. Leukemia (2012) 26:910–7. 10.1038/leu.2011.315 22064349

[96] Stiborova M Eckschlager T Poljakova J Hrabeta J Adam V Kizek R The synergistic effects of DNA-targeted chemotherapeutics and histone deacetylase inhibitors as therapeutic strategies for cancer treatment. Curr Med Chem (2012) 19:4218–38. 10.2174/092986712802884286 22680633

[97] Tassara M Dohner K Brossart P Held G Gotze K Horst HA Valproic acid in combination with all-trans retinoic acid and intensive therapy for acute myeloid leukemia in older patients. Blood (2014) 123:4027–36. 10.1182/blood-2013-12-546283 24797300

[98] Ryningen A Stapnes C Lassalle P Corbascio M Gjertsen BT Bruserud O. A subset of patients with high-risk acute myelogenous leukemia shows improved peripheral blood cell counts when treated with the combination of valproic acid, theophylline and all-trans retinoic acid. Leuk Res (2009) 33:779–87. 10.1016/j.leukres.2008.10.005 19007987

[99] Skavland J Jørgensen KM Hadziavdic K Hovland R Jonassen I Bruserud O Specific cellular signal-transduction responses to in vivo combination therapy with ATRA, valproic acid and theophylline in acute myeloid leukemia. Blood Cancer J (2011) 1:e4. 10.1038/bcj.2011.2 22829110 PMC3255270

[100] Ehsanian R Van Waes C Feller SM Beyond DNA binding – a review of the potential mechanisms mediating quinacrine’s therapeutic activities in parasitic infections, inflammation, and cancers. Cell Commun Signal (2011) 9:13 10.1186/1478-811X-9-13 21569639 PMC3117821

[101] Gurova KV Hill JE Guo C Prokvolit A Burdelya LG Samoylova E Small molecules that reactivate p53 in renal cell carcinoma reveal a NF-kappaB-dependent mechanism of p53 suppression in tumors. Proc Natl Acad Sci U S A (2005) 102:17448–53. 10.1073/pnas.0508888102 16287968 PMC1297696

[102] Eriksson A Österroos A Hassan S Gullbo J Rickardson L Jarvius M Drug screen in patient cells suggests quinacrine to be repositioned for treatment of acute myeloid leukemia. Blood Cancer J (2015) 5:e307. 10.1038/bcj.2015.31 25885427 PMC4450329

[103] Guzman ML Neering SJ Upchurch D Grimes B Howard DS Rizzieri DA Nuclear factor-kappaB is constitutively activated in primitive human acute myelogenous leukemia cells. Blood (2001) 98:2301–7. 10.1182/blood.V98.8.2301 11588023

[104] Keutgens A Robert I Viatour P Chariot A. Deregulated NF-kappaB activity in haematological malignancies. Biochem Pharmacol (2006) 72:1069–80. 10.1016/j.bcp.2006.06.011 16854381

[105] Fathi AT Chen YB. The role of FLT3 inhibitors in the treatment of FLT3-mutated acute myeloid leukemia. Eur J Haematol (2017) 98:330–6. 10.1111/ejh.12841 28000291

[106] Taylor SJ Duyvestyn JM Dagger SA Dishington EJ Rinaldi CA Dovey OM Preventing chemotherapy-induced myelosuppression by repurposing the FLT3 inhibitor quizartinib. Sci Transl Med (2017) 9:eaam8060. 10.1126/scitranslmed.aam8060 28794285

[107] Masson K Rönnstrand L. Oncogenic signaling from the hematopoietic growth factor receptors c-Kit and Flt3. Cell Signal (2009) 21:1717–26. 10.1016/j.cellsig.2009.06.002 19540337

[108] Meshinchi S Appelbaum FR. Structural and functional alterations of FLT3 in acute myeloid leukemia. Clin Cancer Res (2009) 15:4263–9. 10.1158/1078-0432.CCR-08-1123 19549778 PMC2716016

[109] Oellerich T Mohr S Corso J Beck J Döbele C Braun H FLT3-ITD and TLR9 use Bruton tyrosine kinase to activate distinct transcriptional programs mediating AML cell survival and proliferation. Blood (2015) 125:1936–47. 10.1182/blood-2014-06-585216 25605370

[110] Lara-Castillo MC Cornet-Masana JM Etxabe A Banus-Mulet A Torrente MA Nomdedeu M. Repositioning of bromocriptine for treatment of acute myeloid leukemia. J Transl Med (2016) 14:261. 10.1186/s12967-016-1007-5 27604463 PMC5015257

[111] Etxabe A Lara-Castillo MC Cornet-Masana JM Banus-Mulet A Nomdedeu M Torrente MA. Inhibition of serotonin receptor type 1 in acute myeloid leukemia impairs leukemia stem cell functionality: a promising novel therapeutic target. Leukemia (2017) 31:2288–302. 10.1038/leu.2017.52 28193998

[112] Siveen KS Uddin S Mohammad RM. Targeting acute myeloid leukemia stem cell signaling by natural products. Mol Cancer (2017) 16:13. 10.1186/s12943-016-0571-x 28137265 PMC5282735

[113] Andresen V Erikstein BS Mukherjee H Sulen A Popa M Sornes S. Anti-proliferative activity of the NPM1 interacting natural product avrainvillamide in acute myeloid leukemia. Cell Death Dis (2016) 7:e2497. 10.1038/cddis.2016.392 27906185 PMC5260983

[114] Mukherjee H Chan KP Andresen V Hanley ML Gjertsen BT Myers AG. Interactions of the natural product (+)-avrainvillamide with nucleophosmin and exportin-1 mediate the cellular localization of nucleophosmin and its AML-associated mutants. ACS Chem Biol (2015) 10:855–63. 10.1021/cb500872g 25531824 PMC4652655

[115] Moschoi R Imbert V Nebout M Chiche J Mary D Prebet T Protective mitochondrial transfer from bone marrow stromal cells to acute myeloid leukemic cells during chemotherapy. Blood (2016) 128:253–64. 10.1182/blood-2015-07-655860 27257182

[116] Peled A Tavor S. Role of CXCR4 in the pathogenesis of acute myeloid leukemia. Theranostics (2013) 3:34–9. 10.7150/thno.5150 23382784 PMC3563079

[117] Coles AJ Cohen JA Fox EJ Giovannoni G Hartung HP Havrdova E Alemtuzumab CARE-MS II 5-year follow-up: efficacy and safety findings. Neurology (2017) 89:1117–26. 10.1212/WNL.0000000000004354 28835403 PMC5595276

[118] Varghese AM Howard DR Pocock C Rawstron AC Follows G McCarthy H NCRI CLL Sub-Group. Eradication of minimal residual disease improves overall and progression-free survival in patients with chronic lymphocytic leukaemia, evidence from NCRN CLL207: a phase II trial assessing alemtuzumab consolidation. Br J Haematol (2017) 176:573–82. 10.1111/bjh.14342 28032335

[119] Corsello SM Bittker JA Liu Z Gould J McCarren P Hirschman JE The drug repurposing hub: a next-generation drug library and information resource. Nat Med (2017) 23:405–8. 10.1038/nm.4306 28388612 PMC5568558

[120] Karjalainen R Pemovska T Popa M Liu M Javarappa KK Majumder MM JAK1/2 and BCL2 inhibitors synergize to counteract bone marrow stromal cell-induced protection of AML. Blood (2017) 130:789–802. 10.1182/blood-2016-02-699363 28619982

[121] March-Vila E Pinzi L Sturm N Tinivella A Engkvist O Chen H On the integration of in silico drug design methods for drug repurposing. Front Pharmacol (2017) 8:298. 10.3389/fphar.2017.00298 28588497 PMC5440551

